# Knowledge and Awareness of the General Public on Lung Cancer Screening Modalities and Lung Cancer Preventive Methods in Riyadh, Saudi Arabia

**DOI:** 10.3390/curroncol33030169

**Published:** 2026-03-16

**Authors:** Suha Kaaki, Khalid Alkhani, Omar Aldosari, Zyad Aldosari, Mohammed Alhuqbani, Khalid Nagshabandi, Ahmad W. Hajjar, Sami A. Al-Nassar, Waseem M. Hajjar

**Affiliations:** 1 Department of Surgery, College of Medicine, King Saud University, Riyadh 11472, Saudi Arabia; skaaki@ksu.edu.sa (S.K.); 438105025@student.ksu.edu.sa (K.A.); 438101225@student.ksu.edu.sa (K.N.); snassar@ksu.edu.sa (S.A.A.-N.); 2 Department of Orthopedics, College of Medicine, King Saud University, Riyadh 11472, Saudi Arabia; 438105019@student.ksu.edu.sa (O.A.); zaaldosari@ksu.edu.sa (Z.A.); 438105037@student.ksu.edu.sa (M.A.); 3 College of Medicine, Alfaisal University, Riyadh 11533, Saudi Arabia; awhajjar@alfaisal.edu

**Keywords:** lung cancer, lung cancer screening, smoking, awareness, low-dose computed tomography, risk stratification, health literacy, organized screening, healthcare policy, equity

## Abstract

Lung cancer remains the leading cause of cancer-related mortality globally and is often diagnosed at advanced stages in Saudi Arabia. This cross-sectional study aimed to quantify public awareness and knowledge of lung cancer screening (LCS) using LDCT and identify barriers to its implementation in Riyadh. A validated 24-item questionnaire was administered to 452 participants to assess demographic factors, smoking history, and LCS knowledge. Results revealed that only 30.1% of participants had heard of LCS, and 50.2% demonstrated “poor” knowledge scores (mean score 11.0 ± 4.97). Higher knowledge scores were significantly associated with being female, having a bachelor’s degree or higher, and being a non-smoker. While 78.1% expressed willingness to undergo screening, the most significant barrier was a lack of knowledge about the test (44.1%), followed by concerns regarding radiation exposure (36.1%). Conversely, a healthcare provider’s recommendation was identified as the primary motivator for 53.3% of respondents. These findings highlight a critical “awareness–willingness” gap. While public willingness is high, this should not be misconstrued as systemic preparedness; substantial educational and structural gaps remain that must be bridged before national implementation can be considered feasible. We conclude that while public willingness is high, successful implementation requires a transition toward organized invitation systems and the use of multifactorial risk profiles. Integrating epidemiological evidence with proactive policy design is essential to ensure that the national program avoids systematic under- or over-inclusion and remains effective for all demographics.

## Simple Summary

Lung cancer is a leading cause of death in Saudi Arabia, yet it is often diagnosed at advanced stages when it is harder to treat. This research was suggested to determine if the public is prepared for a national screening program using low-dose computed tomography (LDCT). The authors aimed to measure current awareness levels and identify specific barriers, such as a lack of information or concerns about radiation, that might hinder participation. The findings show that while overall knowledge is low, there is a strong willingness to undergo screening if advised by a healthcare provider. This study provides the evidence-based foundation required for policy-driven identification of high-risk groups and the design of equitable recruitment strategies. By bridging these knowledge gaps, health authorities can move beyond technology alone to establish a nationwide program.

## 1. Introduction

Lung cancer is the leading cause of cancer worldwide, with a yearly incidence of 2.26 million [1]. It is also the most common cause of cancer-related death and disability-adjusted life years (DALY), accounting for 2.04 million deaths and 45.9 million DALYs among both sexes in 2019 [1]. Saudi Arabia had an incidence of 1145 cases and 1001 deaths attributed to lung cancer among both sexes in 2020 [2]. However, these numbers may not fully reflect the current burden, as the lack of a screening program could contribute to an underestimation of the actual incidence, particularly given the increasing prevalence of smoking in Saudi Arabia. The lack of a nationwide lung cancer screening program emphasizes the need to ensure that patients and physicians understand the importance of lung cancer screening in individuals at high risk. In recent years, the leading causes of DALYs have been smoking, air pollution, occupational carcinogens, dietary, metabolic, and other environmental risks [3].

Lung cancer is known for its aggressive nature and lack of noticeable early symptoms, leading to diagnostic and treatment delays, which in turn result in poor outcomes. A retrospective cohort study analyzing lung cancer incidence and epidemiology in Saudis between 2006 and 2016 found that ~60% of lung cancer cases were diagnosed at an advanced stage [4]. This study highlighted an increase in crude incidence rates among Saudis [4] and could be used to help develop future screening and prevention programs in the country.

The NELSON trial showed that among high-risk individuals, the use of LDCT for screening was effective in detecting the early stages of lung cancer [5]. It was conducted after the National Lung Screening Trial (NLST) in the United States [6]. Both trials targeted participants with a significant smoking history. Specifically, the NLST demonstrated a 20% reduction in lung-cancer mortality after a follow-up of 6.5 years [6], while the NELSON trial reported a 25% reduction in lung-cancer mortality among high-risk males at 10 years of follow-up [5]. The differences between the trials were mainly in nodule classification and nodule management protocols. The incidence of false-positive results was lower in the NELSON trial than in the NLST [5,6]. While these trials demonstrate significant mortality reduction, it is crucial to recognize that setting appropriate screening criteria is a delicate balance. Criteria that are too broad may increase costs, false positives, and the risk of overdiagnosis, whereas overly restrictive criteria may fail to identify individuals who could benefit most. Establishing evidence-based thresholds that are regularly updated with regional epidemiological data is therefore essential for a sustainable program.

Currently, guidelines categorize patients for screening using LDCT into high- and low-risk individuals. Individuals aged ≥ 50 years with a smoking history of 20 pack-years or more constitute high-risk individuals. These individuals qualify for lung cancer screening using LDCT. The timing of the subsequent LDCT screening changes depending on the nodule size found in the initial LDCT screening, if any is found [7]. If no nodules were detected, the scan would be repeated annually. The current guidelines issued by the Saudi Lung Cancer Association do not recommend mass screening. However, they provide guidance for healthcare providers considering screening very high-risk individuals [8].

Several barriers to lung cancer screening have been identified. A study in the US found that 37% of eligible patients refused to undergo screening when offered by their healthcare provider [9]. It also found that 38% of eligible individuals were unaware of the need for lung cancer screening. Another study found that 20% of current smokers believe that they have “smoked too long to benefit” from screening programs [9]. Furthermore, patients fear the adverse effects of cancer treatment more than lung cancer itself [10]. Barriers involving healthcare providers have also been studied, which suggests that up to one-third of healthcare providers are unaware of current lung cancer screening guidelines [9]. Our study aimed to quantify the awareness and knowledge gap between the general and high-risk populations while identifying the barriers to implementation. Beyond identifying these gaps, we seek to provide the baseline data necessary for defining evidence-based screening thresholds and policy-driven identification of high-risk groups, which are critical for the design of effective screening programs.

## 2. Materials and Methods

### 2.1. Study Setting and Population

This cross-sectional study was conducted between September 2022 and November 2022 among the adult population of Riyadh, Saudi Arabia. Riyadh residents were approached at a major shopping mall in the city. This convenience sampling method was utilized for its accessibility but is acknowledged as a limitation regarding the representativeness of the general population. Anyone over the age of 16 years who could read, comprehend, and write an appropriate response to the questionnaire qualified as a participant. The inclusion of participants from age 16 upwards allows for a broad assessment of public awareness, though it creates a mismatch with the typical screening-eligible population of those aged 50 and older.

### 2.2. Sample Size Calculation

The sample size was calculated based on the 2017 estimated population size of Riyadh Province of 8,216,284 people [11] with a 5% margin of error and 95% confidence level. The projected proportion was chosen as 50% to increase the number of participants because there is little research on our target proportion, particularly in the region. The final sample comprised 452 participants. More than 500 participants were approached to participate in the study to compensate for refusals and incomplete responses.

### 2.3. Methods

Four hundred and sixty-two participants agreed to participate in the study and were approached by trained medical students using a pre-set questionnaire designed to assess their knowledge of and attitudes toward lung cancer screening. Of them, 452 met the inclusion criteria. The questionnaire used was created based on the expanded health belief model, updated National Comprehensive Cancer Network lung cancer screening guidelines, a literature review, and discussions within the research team [7]. The questionnaire began with informed consent, followed by a set of 23 items structured into four sections. The first section consisted of five questions regarding the demographic characteristics of the participants. The second section contained items related to smoking history. This was followed by a section aimed at evaluating the knowledge and awareness of lung cancer. The last section was aimed at evaluating the knowledge and awareness of lung cancer screening and perceived barriers and facilitators pertaining to screening. The questionnaire was created in English and translated into Arabic by two native Arabic speakers. The first translator had experience in building research instruments, medical terminology, and clinical medicine practice. The second translator had no medical background or prior knowledge of how this research instrument was built. Following agreement among field experts who evaluated both drafts, a final version was adopted. The appropriate language was chosen depending on the participants’ preferences. No names or IDs were collected to protect the participants’ privacy. A limitation of this study is that formal psychometric validation, such as reliability testing of the questionnaire, was not performed.

### 2.4. Ethical Consideration

This study was approved (E-22-7376) by our institutional review board. Participants were informed of the aims and objectives of the study, and informed consent was obtained from all participants after reassuring them that their confidentiality was maintained by the exclusion of any identifying data from the questionnaire. All participants were informed of their right to withdraw from the study at any given time. The participants were assured of the anonymity and privacy of their answers to minimize the potential bias associated with self-reported data.

### 2.5. Statistical Analysis

Knowledge and awareness of lung cancer screening were assessed using a 24-item questionnaire, with correct answers coded as 1 and incorrect answers coded as 0. The items were designed to capture a combination of general lung cancer awareness (symptoms and risk factors) and screening-specific knowledge (eligibility and procedural details). The total knowledge score was calculated by adding the scores for all 24 items. A score ranging from 0 to 24 points was generated; the higher the score, the greater the knowledge of lung cancer screening. The 50% and 75% cutoffs used to categorize knowledge levels were based on similar scoring conventions used in regional health awareness exploratory studies. By using 50% and 75% as the cutoff points to determine the level of knowledge, the participants were categorized as having poor knowledge if the score was <50%, as having moderate knowledge if the score was between 50% and 75%, and as having good knowledge if the score was >75%.

Categorical variables are presented as numbers and percentages, whereas continuous variables are summarized as means and standard deviations. The association between the knowledge score and the sociodemographic and other related characteristics of the participants relative to lung cancer screening was assessed using the Mann–Whitney Z-test. Normality was tested using the Shapiro–Wilk and Kolmogorov–Smirnov tests. Knowledge scores followed a non-normal distribution. Therefore, a nonparametric test was conducted. Furthermore, the relationship between having heard of lung cancer screening in terms of the level of knowledge and agreement to undergo LDCT was determined using the chi-squared test. Statistical significance was set at *p* < 0.05. All data analyses were performed using the Statistical Package for Social Sciences v26 (SPSS, IBM Corp, Armonk, NY, USA).

## 3. Results

Four hundred and fifty-two responses were analyzed. Of the participants, 44.9% were aged between 21 and 30 years, with nearly 60% being male, and approximately half of the participants (50.4%) had bachelor’s degrees. Respondents earning 10,000 (2664.5$) to 20,000 (5329$) Saudi Riyal (SAR) per month constituted 31.2% of the cohort. Additionally, only 9.2% were employed in the medical field; the majority (54.6%) were working in non-medical fields, while the rest were retired, unemployed, or students. Table 1 summarizes the sociodemographic characteristics of the participants.

The prevalence rates of current smokers and ex-smokers in our sample were 23.9% and 5.5%, respectively. Table 2 summarizes the history of tobacco and lung cancer.

**Figure 1:**
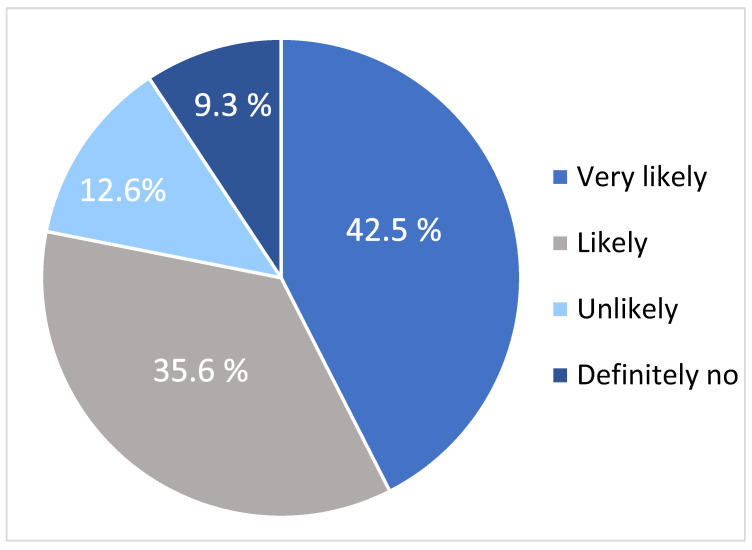
Level of Agreement to Undergo LDCT.

Of them, more than half (54.9%) had started smoking before they turned 18, and 60.2% had been smoking for 10 years or less. Moreover, 34.6% of the current/ex-smokers smoked 11 to 20 cigarettes per day, while 27.1% spent from 201 (53.6$) to 400 (106.7$) SAR per month on smoking. Of those who were previous smokers, 32% reported smoking cessation in the previous 6 months. Of the current smokers, 71.3% planned to stop smoking, and the most common motivation for this was the health risk associated with smoking (61.8%). Among those who did not intend to stop smoking, the most common reasons for this decision were nicotine addiction (29%), being surrounded by smokers (29%), and using smoking as a stress coping mechanism (29%). Regarding the ex-smokers, the most frequently reported aid for smoking cessation was behavioral therapy (32%). The proportion of participants who had heard of lung cancer screening was 30.1%. A family history of lung cancer was reported by 5.5% of the sample. One-third (33.6%) of the respondents believed that the combined efforts of healthcare providers and patients should be responsible for lung cancer screening. Participants’ levels of agreement regarding actions to decrease lung cancer risk and their willingness to undergo LDCT are illustrated in Figure 1.

**Figure 2:**
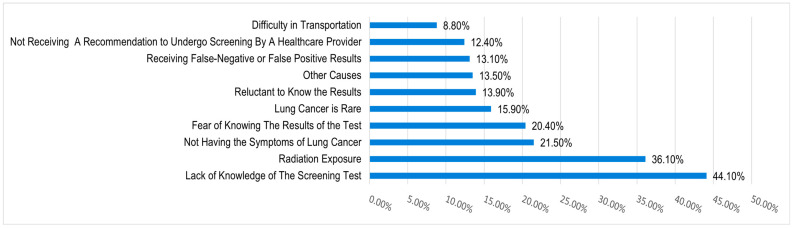
Barriers to undergoing LDCT.

**Figure 3:**
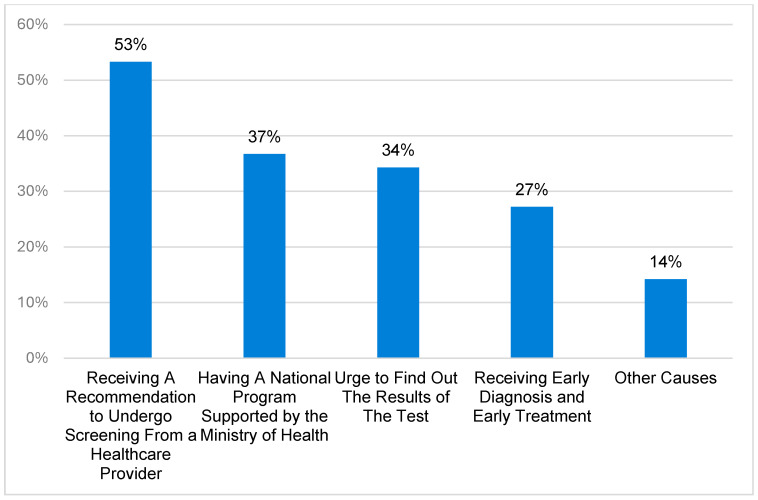
Motivators to Undergo LDCT.

The primary barriers to undergoing LDCT were a lack of screening-specific knowledge (44.1%) and concerns regarding radiation (36.1%) (Figure 2). Conversely, the strongest motivators were healthcare provider recommendations (53.3%) and the potential availability of a government-supported national program (Figure 3).

The participants’ knowledge of lung cancer screening was assessed (Table 3).

In terms of general awareness, participants identified various warning signs of lung cancer, with coughing up blood and persistent cough being the most frequently recognized (Table 3). Most respondents believed that lung cancer could be screened (73.9%), and more than half (54.6%) believed that it could be treated. Only 15.5% were aware that the age group of 50 years and older was at the highest risk of developing lung cancer. The participants believed that the most common factor that increased their chance of developing lung cancer was smoking (83.4%), followed by using electronic cigarettes (57.3%) and a family history of lung cancer (43.8%). The majority of the study population held positive attitudes toward the efficacy of lung cancer screening (Table 3). A high percentage (44%) agreed that lung cancer screening tests are beneficial. However, more than two-thirds also believed that the radiation risk surpasses the benefits of lung cancer screening through LDCT. and only 14.2% knew that the target age group for lung cancer screening was 50 to 75 years. The overall mean knowledge score was 11.0 (SD = 4.97). Poor, moderate, and good knowledge levels were found in 50.2%, 40.7%, and 9.1% of the participants, respectively.

When quantifying the association between the knowledge score in terms of the sociodemographic and other related characteristics of the participants, Table 4 summarizes the association between the knowledge score in terms of the sociodemographic and other related characteristics of the participants. It was found that a higher knowledge score was more likely associated with the female sex (Z = 5.878; *p* < 0.001), having a bachelor’s or higher degree (Z = 4.850; *p* = 0.001), being a non-smoker (Z = 5.868; *p* < 0.001), and an intention to undergo LDCT (Z = 3.269; *p* < 0.001).

No significant association was observed between the knowledge score and age, family monthly income, employment status, knowledge of lung cancer screening, or family history of lung cancer. Participants who claimed to have heard of lung cancer screening were not significantly more likely to have higher knowledge scores for lung cancer screening. Table 5 summarizes the relationship between having heard of lung cancer screening according to the participants’ level of knowledge and agreement to undergo LDCT.

## 4. Discussion

The objective of this study was to evaluate the degree of public awareness and interest in undergoing lung cancer screening in Riyadh, Saudi Arabia. The findings revealed that only 30.1% of participants had heard of lung cancer screening, which is lower than that reported in a study conducted in the USA, which found 52% of participants were aware of the practice. In terms of attitude, 78.1% of our study population was willing to undergo lung cancer screening. This percentage is comparable to that reported in a study conducted in the USA, in which 80.6% of the population was willing to undergo lung cancer screening [12]. It should be noted that the numbers in both our study and previous studies might not reflect real-life willingness to undergo LDCT, because the survey did not mention the specific method used for screening, which participants could have assumed was chest radiography or other less invasive tests compared with LDCT [13]. Similarly, our study also revealed that only 11.8% of those who had heard of lung cancer screening had a thorough understanding of it.

In contrast to research published in the USA that found a correlation between older age and screening consciousness, albeit a statistically insignificant one, the current study found no significant relationship between age and awareness of lung cancer screening [12]. Saudi Arabia has a populace that is considerably younger than that of the USA, with a low estimated prevalence of 3.3% of people older than 65 years of age [14]. In comparison, the USA has an elderly population with a higher percentage of people older than 65 years of age, with the prevalence being 18.5% [15]. Therefore, there may have been a difference in the relationship between age and awareness of lung cancer screening between the two groups. However, these age-related interpretations remain speculative and should be framed as hypothesis-generating. Older people may be more conscious of health risks and precautionary steps, such as lung cancer screening, in the USA, where the population is aging. Age may not be as closely correlated with the awareness of lung cancer screening in Saudi Arabia, which has a younger population. However, it is important to note that this is speculative. Further research is required to confirm the association between age and knowledge of lung cancer screening in different populations. Sharma et al. suggested that there was no difference between men and women in the proportion of people who had heard of lung cancer screening [12]. However, the present study revealed a statistically significant difference in knowledge scores between the sexes, with female participants scoring more than 2.5 points higher on the 24-point knowledge score than male participants. The noted variations in findings could have been caused by differences in survey methods or group characteristics between the two studies. According to the current research, better education is related to greater awareness of lung cancer screening, which is consistent with published literature. However, there was no correlation between income and awareness of lung cancer screening, which is consistent with the published literature [12]. However, comparisons with U.S. data must be made cautiously, given the fundamental differences in healthcare infrastructure, insurance models, and the long-standing availability of established screening programs that are not yet present in Saudi Arabia.

Our study showed that the most common barrier toward lung cancer screening was “lack of knowledge about the screening test,” with 44.2% of the sample agreeing that this was a barrier. This is consistent with research on awareness among primary care physicians, where one of the most commonly perceived barriers was a lack of awareness by patients [16]. Furthermore, “radiation hazard” was also a perceived barrier by most participants, both in this study and in previous studies [16]. More than half (53.3%) of the participants in this study believed that receiving a recommendation from a healthcare provider would motivate them to undergo screening tests. This highlights that recruitment success is tied to clear risk communication and shared decision-making. Because passive recruitment approaches often favor individuals with higher health literacy, a shift toward organized invitation systems is necessary to ensure equitable coverage across different socioeconomic strata in Riyadh. This finding is consistent with those of studies on the perspectives of the general population and primary care physicians. This is particularly significant, because previous studies assessing physicians’ awareness showed low awareness, which is the strongest motivator for participants to undergo LDCT. However, increasing awareness is only half the battle; the success of recruitment strategies also depends on fostering deep-rooted trust in the healthcare system. One-third of the participants in the current study believed that a national screening program supported by the Ministry of Health was a motivator to undergo LDCT. It is noteworthy that within the demography of this sample, no screening program was available as of writing this manuscript. This contrasts with studies reported in the literature, in that these studies were conducted in regions with a national screening program. Strengths of our study include having a large sample size, thorough translation and validation of the tool, and data collection from large social areas, attracting a diverse sample of the population. While the authors developed the tool following standard measures, a limitation of the study was that it was the first attempt to administer the tool on a large sample. Another limitation was taking data collection from only one city. Furthermore, our reliance on convenience sampling in a single city and the resulting young cohort (88.3% <40 years) are significant limitations that restrict the generalizability of our findings to the high-risk, screening-eligible population. Additionally, while detailed smoking history was collected, specific pack-year exposures were not calculated, which further limited the assessment of true screening eligibility within this sample.

This study revealed that more than half of participants had poor knowledge of lung cancer screening. Female participants, individuals with a higher educational level, and nonsmokers achieved higher knowledge scores than those of male participants, individuals with lower educational attainment, and smokers, respectively. The findings also indicated that greater knowledge of lung cancer screening was associated with a higher likelihood of intending to undergo LDCT screening. These results underscore the significance of targeted education and awareness campaigns, particularly among high-risk individuals, to enhance their knowledge about lung cancer screening.

### Clinical Significance

While the efficacy of LCS using LDCT is well-established globally [17], its successful implementation in Saudi Arabia depends on population readiness. The clinical significance of this study lies in identifying a critical “awareness–willingness” gap that clinicians can actively bridge. Our data reveal that while public knowledge of LCS is poor (50.2%), the willingness to undergo screening is high (78.1%) when recommended by a healthcare provider.

This identifies a vital window for clinical intervention: patients are unlikely to request screening independently due to a lack of awareness, yet they are highly responsive to physician guidance. For healthcare providers, this underscores the necessity of initiating risk assessments and LCS discussions during routine visits for high-risk individuals (smokers aged ≥50), rather than waiting for patient inquiry. Furthermore, because “lack of knowledge” was the most reported barrier (44.1%), clinicians must prioritize point-of-care education to mitigate unfounded fears regarding radiation and clearly explain the survival benefits. As suggested by Wu and Chang, integrating multifactorial risk profiles—including environmental exposures and population-specific characteristics—is essential for the precise recruitment and sustainability of screening initiatives, particularly in diverse Asian populations [18]. It is therapeutically critical to bridge this information gap, as around 60% of lung cancer cases in Saudi Arabia are being identified at an advanced stage [4].

## 5. Conclusions

Participants who have heard of LCS are more likely to undergo it; therefore, further efforts are needed to increase the awareness of smokers and past smokers about the availability of the LCS test. This should come in parallel with efforts to encourage physicians to offer an LDCT screening test to eligible candidates. And while this study aimed at studying the general population’s knowledge of LDCT as a lung cancer screening tool, studying physician/healthcare provider knowledge and practices is an equally essential part of anticipating the needs of a lung cancer screening program. Furthermore, as the transition toward a national program begins, shifting from passive self-referral to organized invitation systems that utilize the multifactorial risk stratification will be necessary to ensure equitable coverage and overcome the barriers identified in this study. In conclusion, while public willingness provides a strong foundation, success is ultimately determined by how well criteria are defined, how risk is conceptualized in policy, and how eligible individuals are successfully engaged through organized, evidence-based systems. Integrating epidemiological evidence, health literacy considerations, and proactive policy design is essential for achieving a precise and sustainable lung cancer screening program in Saudi Arabia. Increasing public awareness of lung cancer screening and educating healthcare providers about its importance are crucial steps in reducing lung cancer-related deaths. Efforts should be made to bridge knowledge gaps, promote physician recommendations, and establish national screening programs.

## Figures and Tables

**Table 1:** The sociodemographic characteristics of the participants.

Study Data	*N* (%)
**Age group**	-
≤20 years	57 (12.6%)
21–30 years	203 (44.9%)
31–40 years	139 (30.8%)
>40 years	53 (11.7%)
**Sex**	-
Male	263 (58.2%)
Female	189 (41.8%)
**Educational level**	-
High school or below	119 (26.3%)
Diploma	65 (14.4%)
Bachelor’s degree	228 (50.4%)
Master’s degree	36 (8.0%)
PhD	4 (0.9%)
Family monthly income (SAR) *	-
<5000	104 (23.0%)
5000–9999	131 (29.0%)
10,000–20,000	141 (31.2%)
>20,000	76 (16.8%)
**Employment status**	
Unemployed	94 (20.8%)
Working in a non-medical field	247 (54.6%)
Working in a medical field	42 (9.3%)
Student	65 (14.4%)
Retired	4 (0.9%)

* 1$ = 3.75 SAR.

**Table 2:** History of tobacco and lung cancer.

Variables	*N* (%)
**Smoking status**	
Non-smoker	319 (70.6%)
Current smoker	108 (23.9%)
Ex-smoker	25 (5.5%)
**At what age did you start smoking?** (*n* = 133)	
<18 years	73 (54.9%)
≥18 years	60 (45.1%)
**Duration of smoking** (*n* = 133)	
≤10 years	80 (60.2%)
>10 years	53 (39.8%)
**If you are a current or past smoker, how many cigarettes did you smoke per day?** (*n* = 133)	
1–5	14 (10.5%)
6–10	32 (24.1%)
11–20	46 (34.6%)
21–30	32 (24.1%)
31–40	5 (3.8%)
>40	4 (3.0%)
How much do you spend per month on tobacco products (SAR) * (*n* = 133)	
100–200	25 (18.8%)
201–400	36 (27.1%)
401–600	31 (23.3%)
601–800	19 (14.3%)
>800	22 (16.5%)
**If you are a previous smoker, when did you stop smoking?** (*n* = 25)	
<6 months	8 (32.0%)
6–12 months	10 (40.0%)
Within the last 5 years	2 (8.0%)
5–10 years	3 (12.0%)
11–15 years	2 (8.0%)
**Do you plan on quitting smoking?** (*n* = 108)	
Yes	77 (71.3%)
No	31 (28.7%)
**What motivates you to stop smoking?** (*n* = 77) ^†^	
Home or office smoking ban	22 (28.6%)
Financial burden	17 (22.1%)
Offensive odor	26 (34.2%)
Health risks	47 (61.8%)
Other causes	11 (14.5%)
**What prevents you from stopping smoking?** (*n* = 31)	
Price of smoking cessation aids/programs	7 (22.5%)
Fear of weight gain	2 (6.5%)
Losing a stress coping mechanism	9 (29.0%)
Surrounded by smokers	9 (29.0%)
Nicotine addiction	9 (29.0%)
**What helped you in quitting smoking?** (*n* = 25)	
Nicotine patches	1 (4.0%)
Smoking cessation medication	1 (4.0%)
Behavioral therapy	8 (32.0%)
Electronic cigarettes, shisha, vaping	3 (12.0%)
Support groups	1 (4.0%)
**Heard of lung cancer screening?**	
Yes	136 (30.1%)
No	316 (69.9%)
**Family history of lung cancer**	
Yes	25 (5.5%)
No	427 (94.5%)
**Who is responsible for lung screening tests?**	
Patient themselves	128 (28.3%)
Healthcare system	48 (10.6%)
Combined effort	152 (33.6%)
Not sure	124 (27.5%)
**To what degree do you agree with this statement: “There are things I can do to decrease my chances of being diagnosed with lung cancer”?**	
Strongly agree	259 (57.3%)
Agree	107 (23.7%)
Disagree	14 (3.1%)
Strongly disagree	8 (1.8%)
I don’t know.	64 (14.2%)

* 1$ = 3.75 SAR. ^†^ Variable with multiple response answers.

**Table 3:** Assessment of general lung cancer awareness and screening-specific knowledge.

General Awareness vs. Screening-Specific Knowledge Items	*N* (%)
**Which of the following are warning signs of lung cancer?**	
Coughing blood [yes]	264 (58.4%)
Persistent worsening cough (>8 weeks) [yes]	236 (52.2%)
Shortness of breath [yes]	229 (50.7%)
Chest pain [yes]	212 (46.9%)
Fatigue [yes]	188 (41.6%)
Loss of appetite [yes]	185 (40.9%)
Hoarseness [yes]	174 (38.5%)
Recurrent lung infections [yes]	223 (49.3%)
Shoulder pain [yes]	95 (21.0%)
Persistent cough for 2–3 weeks [yes]	160 (35.4%)
**General Knowledge**	
Can lung cancer be screened? [yes]	334 (73.9%)
Can lung cancer be treated? [yes]	247 (54.6%)
Which age group is at the highest risk of developing lung cancer? [≥50 years]	70 (15.5%)
**Which of the following increases the chance of developing lung cancer?**	
Smoking [yes]	377 (83.4%)
Electronic cigarettes [yes]	259 (57.3%)
Family history of lung cancer [yes]	198 (43.8%)
Secondhand smoking [yes]	179 (39.6%)
Air pollution [yes]	167 (36.9%)
Occupational exposure (asbestos, nickel, chromium) [yes]	140 (31.0%)
Radon gas exposure [yes]	106 (23.5%)
Asthma [yes]	55 (12.2%)
Overweight/obesity [yes]	42 (9.3%)
Being under pressure/stress [yes]	35 (7.7%)
Vitamin supplements [yes]	15 (3.3%)
Infections and inflammations [yes]	61 (13.5%)
Sedentary lifestyle [yes]	56 (12.4%)
Use of mobile phone [yes]	9 (2.0%)
Lung diseases (COPD *, fibrosis) [yes]	196 (43.4%)
Poor eating habits (fast food) [yes]	82 (18.1%)
**Attitudes toward screening**	
LCS tests can detect cancer before symptoms appear [agree].	233 (51.5%)
LCS tests save lives [agree].	285 (63.1%)
LCS tests can detect cancer in treatable stages [agree].	300 (66.4%)
Radiation from lung cancer screening tests can cause further damage [disagree].	128 (28.3%)
There is no benefit from lung cancer screening tests [disagree].	253 (56.0%)
**Target groups**	
Target group [Smokers or past smokers > 20 years and age ≥ 50]	165 (36.5%)
Target age group in the screening of lung cancer (50–75 years old)	64 (14.2%)
Total knowledge score (mean ± SD)	11.0 ± 4.97
**Level of knowledge**	
Poor	227 (50.2%)
Moderate	184 (40.7%)
Good	41 (9.1%)

* Chronic obstructive pulmonary disease.

**Table 4:** Association between the knowledge score and the sociodemographic and other related characteristics of participants relative to lung cancer screening.

Factor	Knowledge Score (24) Mean ± SD	Z-Test	*p*-Value ^§^
**Age group**			
<30 years	11.0 ± 4.99	0.405	0.685
≥30 years	11.1 ± 4.94		
**Sex**			
Male	9.92 ± 5.07	5.878	**<0.001** **
Female	12.6 ± 4.38		
**Educational level**			
Diploma or below	9.69 ± 4.69	4.850	**<0.001** **
Bachelor’s or higher	11.9 ± 4.94		
Family monthly income (SAR) *			
<10,000	10.8 ± 4.81	0.806	0.420
≥10,000	11.3 ± 5.13		
**Employment status**			
Unemployed	11.1 ± 4.50	0.054	0.957
Employed	11.0 ± 5.22		
**Smoking status**			
Non-smoker	11.9 ± 4.84	5.868	**<0.001** **
Smoker/Ex-smoker	8.90 ± 4.62		
**Heard of lung cancer screening**			
Yes	11.8 ± 4.81	1.916	0.055
No	10.7 ± 5.00		
**Family history of lung cancer**			
Yes	11.5 ± 4.93	0.378	0.705
No	11.0 ± 4.97		
**Agreement to undergo LDCT**			
Very likely/Likely	11.5 ± 4.85	3.269	**0.001** **
Unlikely/Definitely no	9.55 ± 5.09		

**^§^** *p*-value has been calculated using Mann–Whitney Z-test. * 1$ = 3.75 SAR. ** Significant at *p* < 0.05 level.

**Table 5:** Relationship between having heard of lung cancer screening according to the participants’ level of knowledge and agreement to undergo LDCT.

	Heard of Lung Cancer Screening		X2	*p*-Value ^§^
Factor	Yes *N* (%) (*n* = 136)	No *N* (%) (*n* = 316)		
**Level of knowledge**			3.555	0.169
Poor	60 (44.1%)	167 (52.8%)		
Moderate	60 (44.1%)	124 (39.2%)		
Good	16 (11.8%)	25 (7.9%)		
**Agreement to undergo LDCT**			0.478	0.489
Very likely/Likely	109 (80.1%)	244 (77.2%)		
Unlikely/Definitely no	27 (19.9%)	72 (22.8%)		

^§^ *p*-value has been calculated using the Chi-square test.

## Data Availability

The data presented in this study are available on request from the corresponding author due to privacy and ethical restrictions.

## Abbreviations

The following abbreviations are used in this manuscript:

DALY	disability-adjusted life years
LCS	lung cancer screening
LDCT	low-dose computed tomography
NLST	National Lung Screening Trial
SAR	Saudi Riyal
